# Kenaf Composites for Automotive Components: Enhancement in Machinability and Moldability

**DOI:** 10.3390/polym11101707

**Published:** 2019-10-17

**Authors:** Nabilah Afiqah Mohd Radzuan, Nur Farhani Ismail, Mohd Khairul Fadzly Md Radzi, Zakaria Bin Razak, Izdihar Binti Tharizi, Abu Bakar Sulong, Che Hassan Che Haron, Norhamidi Muhamad

**Affiliations:** 1 Centre for Materials Engineering and Smart Manufacturing, Faculty of Engineering and Built Environment, Universiti Kebangsaan Malaysia, Bangi 43600, Selangor, Malaysia; afiqah@ukm.edu.my (N.A.M.R.); farhani@gmail.com (N.F.I.); mkfadzly@gmail.com (M.K.F.M.R.); zakaria@gmi.edu.my (Z.B.R.); izdihar@gmail.com (I.B.T.); chase@ukm.edu.my (C.H.C.H.); norhamidi@ukm.edu.my (N.M.); 2 Unit of Farm Mechanisation, Biological Research Division, Malaysian Palm Oil Board (MPOB), Bangi Lama, Kajang 43000, Selangor, Malaysia; 3 Department of Production Technology, German-Malaysian Institute, Taman Universiti, Kajang 43000, Selangor, Malaysia

**Keywords:** Fiber/matrix bond, mechanical properties, Mechanical testing, compression moulding

## Abstract

To date, the mechanical performance of kenaf composites is still unsatisfied in term of its mechanical performance. Therefore, research focuses on kenaf composites fabrication through the selection of polymer resin, including epoxy, polypropylene, and polylactic acid. The incorporated kenaf fibre at 10 wt % to 40 wt % loadings was conducted using injection and a compression moulding process. The compressed materials indicated high tensile strength at 240 MPa compared to inject materials (60 MPa). Significant improvement on impact strength (9 kJ/m^2^) was due to the unpulled-out fibre that dispersed homogenously and hence minimize the microcrack acquire. Meanwhile, high flexural strength (180 MPa) obtained by kenaf/epoxy composites due to the fibre orientate perpendicular to the loading directions, which improve its mechanical properties. The findings indicate that the kenaf fibre reinforced thermoset materials exhibit better mechanical properties as a function to the battery tray applications.

## 1. Introduction

Polymers composite materials have attracted the attention of numerous researchers and industries as it can easily apply to a wide range of applications. These include automotive parts, constructions, building, manufacturing, military, and many other engineering fields [1,2,3]. These interests rise since composite materials have become an alternative to the conventional product available in today’s market. Composite materials offer much lower costs, are less time-consuming, and their performance can be controlled based on application needs [4,5]. Automotive industries now provide more advanced features like the latest technologies inventions due to the demand required. On the other hand, these composite materials usually embedded as a components such as battery tray, engine cover and else which contribute consumer-preferred vehicles that offer energy saving and green technology [6,7,8]. This rapid growth in the use of natural fibres like kenaf composites over conventional materials is undeniable as they possess excellent mechanical properties [7,9,10].

Kenaf fibre offers many added advantages in term of low density with higher specific strength and stiffness [11]. Also, it benefits in term of low hazard during the manufacturing processes and low emissions of toxic fumes when subject to heat. Moreover, it also reciprocates oxygen to the environment, is a renewable fibre source, and has the benefit of lower production costs [12,13,14]. Based on the knowledge of kenaf fibre, researchers tend to incorporate it with polymer resin due to the resulting substantial change in mechanical performances. However, studies report that the main drawback of kenaf composite is poor compatibility between the fibre and the polymer. Also, high humidity deteriorates the structure of the composite as the microcrack emerge, lowering its mechanical strength [3,15,16]. Surface treatments are an approach used to minimise the incompatibility gap between hydrophobic and hydrophilic materials. These treatments modify the fibre structure, which repairs the mechanical bonding to the polymers. Thus, researchers have focused on improving and investigating which polymer is suitable to be used in kenaf composites for both thermosets and thermoplastics [10,17].

Others than the materials selected, the manufacturing process and machinability seems crucial for their ability to enhance overall performance [6,18]. The manufacturing process, in term of injection moulding and compression moulding, is often discussed as it aids in dispersion and interparticle distance between the composite matrix [6,19]. Thus, different manufacturing process able to produce the different performance of composite materials. Even though the materials used and processing method are perfectly adequate, there are still problems with the kenaf fibres as their mechanical properties underperform especially at temperature above 80 °C [3,20,21]. Thus, studies on the materials and processing method have discussed in detail the balance between mechanical properties and the complex behaviour of the kenaf fibre. Hence, this study focused on the mechanical properties and morphology of the kenaf composites to develop an advanced composite used in automotive applications such as the battery tray.

## 2. Experimental

### 2.1. Materials 

**Figure 1:**
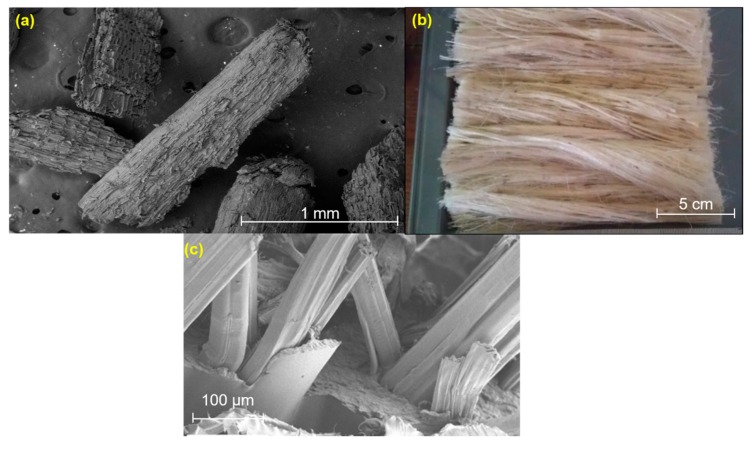
The different types of kenaf fibre (**a**) kenaf mesh core filler, (**b**) kenaf fibre (bio-retting), and (**c**) kenaf composite.

Two types of polymer were used, including thermoplastic and thermoset materials, which are polypropylene, maleated polypropylene (MAPP), polylactic acid, and epoxy resin. A polypropylene (PP) impact copolymer for automotive application was supplied by Lotte Chemical TITAN (Kuala Lumpur, Malaysia) in the form of pallet commercially known as PP grade SM850, which consists of 0.9 g/m^3^ and has mass flow rate of 45 g/10 min. Meanwhile, polylactic acid (PLA) supplied by Shenzhen Esun Industrial Co., Ltd., Shenzhen, China was in pallet form with a density of 1.25 g/cm^3^ and a melt flow rate of 12 g/10 min. Also, the liquid epoxy resin grade D.E.R 331 was supplied by the Dow Chemical Company (“Dow Chemical Pasific Ltd.”) (Petaling Jaya, Malaysia), with a density of 1.16 g/cm^3^ and mass flow rate of 0.05 g/min. Epochemie International Pte Ltd. (Woodlands Bizhub, Singapore) supplied the curing agent NaOH at 6 wt.% treatment (JOINTMINE TM 925-3S). The kenaf, which is a herbaceous plant, was supplied by Lembaga Kenaf & Tembakau Negara (LKTN) (Kelantan, Malaysia). It consists of various parts including kenaf bast, core, and kenaf fibre (bio-retting). The sizes of the kenaf bast and core are between 160 µm to 900 µm, as shown in Figure 1a, at different mesh sizes—20 mesh core filler and 40 mesh core filler. The kenaf fibre (bio-retting) possess longer fibres (17.5 cm as in Figure 1b). The addition of carbon nanotubes (CNT) was used as an additive in this study. It was supplied by Nanocyl Belgium (Sambreville, Belgium) and had a surface area of 300 m^2^/g and diameter of 9.5 nm with a length of >1.5 µm [22]. Figure 1c exhibited the actual micrograph images of the kenaf composites. The kenaf bast and core are composed of cellulose, hemicellulose, and lignin [13]. Also, Figure 1c indicated that the kenaf composites could be easily pulled out as the resin did not correctly hold the kenaf fibres. These phenomena will weaken the overall performance of kenaf composites.

### 2.2. Fibres Preparation

The two primary fabrication processes included injection moulding and a compression moulding process in which the kenaf composites was initially prepared using an internal mixer and mechanical stirrer. The kenaf core and kenaf bast–reinforced polypropylene resin were mixed using a Sigma Blade mixer at a temperature of 185 ℃ and a speed of 45 rpm for 25 minutes. The compositions of the kenaf fibre were between 10 wt % to 40 wt %. Meanwhile, for studies using an additive, compositions of 1 wt % to 4 wt % CNT were mixed with 30 wt % kenaf fibre. For kenaf fibre (bio-retting) preparations, the kenaf was dried in the oven for 5 minutes to remove the fibre moisture. Meanwhile, for thermoset material preparations, the kenaf fibre was soaked in 6 wt % NaOH solution for 24 hours before being immersed in 1 wt % acetic acid glacial for 5 minutes. The kenaf fibre was soaked in distilled water for 30 minutes and washed until the kenaf fibre had a pH of 7 before being dried in the oven at 40°C for another 24 hours. Finally, the kenaf fibre was mixed with epoxy resin and hardener using the mechanical stirrer (model KIKA 20 RW) at a rotational speed of 2 rpm for 5 minutes.

### 2.3. Method of Fabrications

The kenaf composite was prepared using the melt compounding method. The feedstock of kenaf bast and core-reinforced polypropylene underwent a crushing process to obtain a pellet size with an average diameter of 3 mm. The feedstock was then injected using the Battenfeld Injection Molding Machine (Universiti Kebangsaan Malaysia, Malaysia)at a processing temperature of 185 °C, an injection rate of 20 cm^3^/s, and an injection pressure and holding pressure of 2000 bar to 2100 bar [23]. The kenaf composite cannot withstand temperatures greater than 185 °C, above which the kenaf fibre will start to degrade. Apart from that, the kenaf fibre–reinforced CNT and polypropylene underwent the same procedure with an injection temperature between 180 °C to 195 °C, injection rate between 10 cm^3^/s to 20 cm^3^/s, and an injection pressure and holding pressure of 1700 bar 1900 bar and 1800 bar to 2000 bar, respectively. The addition of CNT additives varied from 1 wt % to 4 wt %, which enabled us to increase the temperature from 185 °C to 190 °C, as the CNT can withstand such a rise in temperature. For kenaf fibre (bio-retting)–reinforced PLA, the composite was hot pressed using the compression moulding at a temperature of 190 °C and a pressure of 5 MPa for 15 minutes at various of compositions—30 wt %, 40 wt %, 50 wt %, and 60 wt %. Meanwhile, the compression moulding technique was also applied to the kenaf fibre–reinforced epoxy resin at a temperature of 75 °C to 125 °C and a pressure of 6 MPa to 10 MPa for 15 to 35 minutes [24]. 

### 2.4. Characterisation of Kenaf Composites

The tensile strength test was conducted at room temperature with a crosshead speed of 1 mm/min using a Instron 5567 Universal Testing Machine (UTM). The produced specimen exhibits a dog-bone shape with an overall length of 165 mm, a distance of 115 mm between the grips, and a gage length of 50 mm (referred to as ASTM D638-03). Meanwhile, for the flexural strength, the tested was performed using the Instron 5576 Universal Testing Machine (UTM) based on the ASTM D790-99 standard with a crosshead speed of 1 mm/min. The flexural strength samples were prepared in the form of a rectangular specimen, 127 mm in length, and with a span length of 120.6 mm. Also, the Instron 5567 Universal Testing Machine was attached to the extensometer used to measure strain in which Young’s modulus *E* is measure based on Equation (1).

(1) $$E=\frac{FL_{0}}{A_{0}\Delta L}$$

where *F* is load in applied force, *L*_0_ is the initial length, *A*_0_ is the square area, ΔL is the changed in length, and *E* is Young’s modulus. Five specimens were used for each test. The impact test was performed using the Izod Impact Strength Test using the ASTM D256 at room temperature. The rectangular shaped specimens were prepared with a notch of 22.5°, a length of 64 mm, a width of 12.7 mm, and a span centre of 32 mm. For this impact test, there were a limited number of specimens used, so not all of the kenaf composites, mesh-types, kenaf/PLA/PP, and CNT were tested. The FESEM analysis was conducted using the Quanta FEI Quanta 400F, the maximum magnification of which is 10 kV. 

## 3. Results and discussion

### 3.1. Tensile Properties 

**Figure 2:**
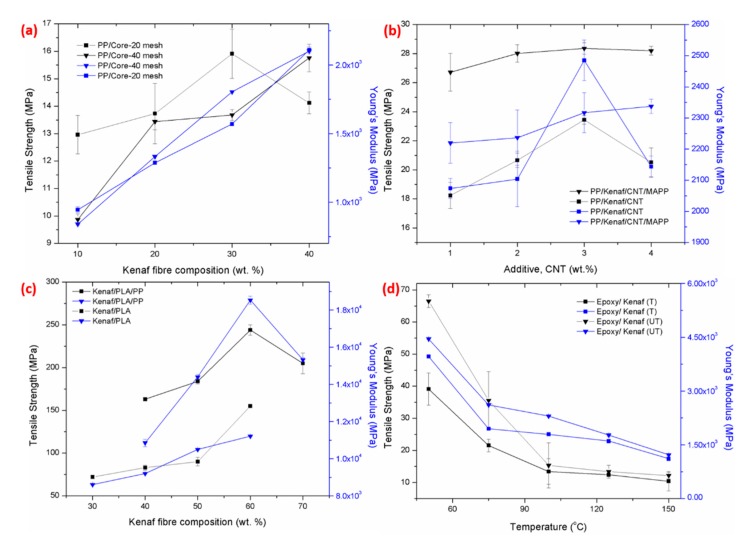
Tensile strength of kenaf composite at different (**a**) kenaf fibre compositions; mesh types, (**b**) additive loading; CNT, (**c**) kenaf fibre compositions; PLA/PP, and (**d**) temperature.

The tensile properties of the kenaf composite were recorded in Figure 2 for various types and compositions of kenaf fibre. Figure 2a demonstrate that the kenaf fibre with a 20 mesh core filler at 30 wt % composition possessed a higher tensile strength at 16 MPa compared to other kenaf fibres. This was due to stronger filler bonding with the resin interfacial adhesion. This further affected and strengthened the kenaf composite, which contrasted with the 40 mesh core filler, which exhibited tensile strength of 13.6 MPa at 30 wt.%. However, as the filler loading increased up to 40 wt %, the tensile strength of the 40 mesh core filler increase drastically to 15.5 MPa, which was in agreement with other reported studies of kenaf composites [14,20,25]. This phenomenon occurred due to the larger surface areas of the filler, which leaves more reactive surfaces for the matrix. These allowed the polymer resin matrix to attach within the kenaf fibre surface, which strengthened the bonding between the fibre and the matrix. The tensile strength of the 20 mesh core filler at 30 wt % is higher compare the rest of the materials. The drop of the tensile strength at 20 mesh was because the composite had a weak interfacial interaction and there was de-bonding between the fibre and the matrix [25,26]. These contributed to the void formation, which lowered the tensile strength due to the quick propagation of cracks [27,28].

The Young’s modulus of the kenaf composite increased with the increase of fibre loading, as reported in Figure 2a. These results demonstrate that the composite was able to withstand a larger load and construct a stiffer composite material [3,25]. Other studies have suggested that the strength of the composite material depends on the properties of the reinforced materials, the matrix used, and the volume fraction of the fibre applied [29]. Figure 2b demonstrates that the tensile strength value of PP/kenaf/CNT/MAPP was higher at 28 MPa compared to PP/Kenaf/NT at 22 MPa. The higher tensile strength obtained was due to the fact that the MAPP acted as a coupling agent, which increased the requirement for interfacial stress to the kenaf fibre [12]. Moreover, the CNT tended to agglomerate the results of a lack of secondary filler distributions [22].

In the prior studies of thermoplastic and thermoset materials, it appears that the thermoset materials exhibit better in mechanical properties as compared to thermoplastic materials [30,31]. Figure 2c indicated that the kenaf composite–reinforced PP/PLA resin encountered the highest tensile strength with 245 MPa compared to kenaf composite–reinforced epoxy resin. Kenaf/PP/PLA composites prepared at 150 °C showed a lower tensile strength with 200 MPa due to significant yield behaviour, which demonstrated a ductile fracture [32]. These are confirmed by the previous studies, which reported that the PLA materials are brittle because they consist of amorphous phases when the temperature rises between 140 °C to 180 °C [32,33]. For epoxy composites, the tensile strength decreased as the kenaf fibres loading increased, predominantly because of the poor matrix adhesion and unbonding between the matrix and fibres [34]. At higher fibre loadings, the fibres tends to overshadow the epoxy resin, which further defects the composites [1,10]. According to studies conducted using kenaf/epoxy materials, as the fibres increased above 15 wt %, the treated composites were unable to act as a surface coating due to the failure in the formation of the mechanical interlocked coating. This phenomenon is shown in Figure 2c, where the untreated kenaf composite acquired 70 MPa, while the treated kenaf obtained 40 MPa at 30 wt % of kenaf loadings.

### 3.2. Flexural Properties

**Figure 3:**
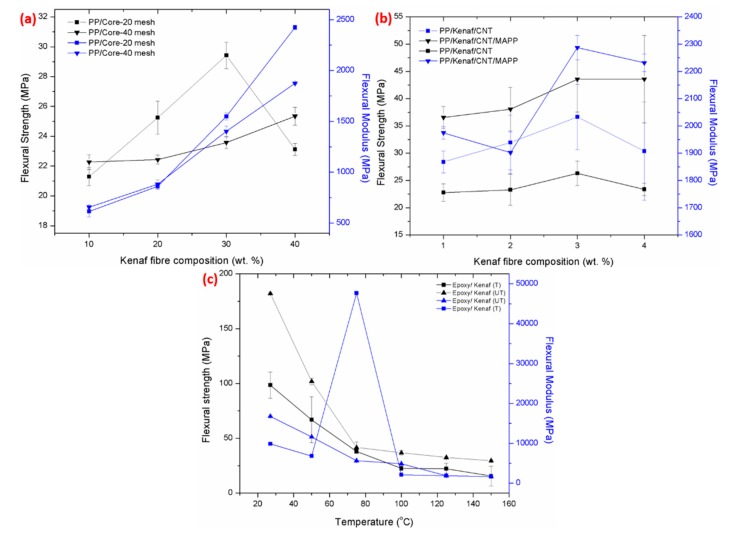
Flexural strength of kenaf composite at different (**a**) kenaf fibre compositions; mesh types, (**b**) additive loading; CNT, and (**c**) temperature.

**Figure 4:**
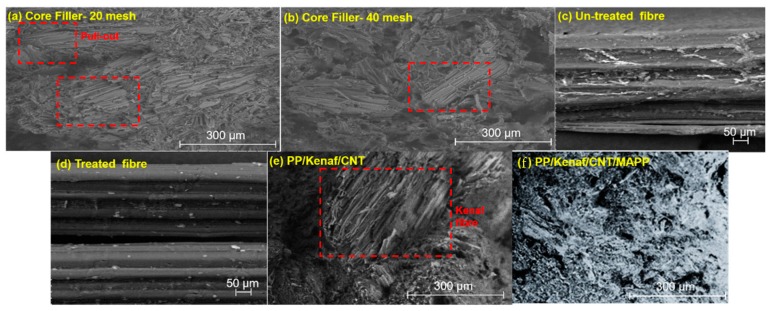
Analysis of kenaf composites.

The flexural strength and the flexural modulus of different kenaf composites and processes are presented in Figure 3 for better comparison. Figure 3a shows the flexural strength of kenaf core-20 mesh reinforced PP that dropped drastically at 40 wt % of kenaf fibres loading with 22 MPa compared to rest of the kenaf composites. These results suggested that, at a higher loading, the kenaf fibres tend to agglomerate and pull out of the bundle, which lowers the mechanical properties, as demonstrated in Figure 4a. These phenomena also cause the kenaf core-20 mesh to exhibit the lowest flexural modulus at 1.4 × 10^3^ MPa. The flexural strength was increased up to 30 MPa for PP/kenaf core-40 mesh due to the better dispersion and excellent adhesion bonding of the fibre matrix [20,34,35]. Figure 4b demonstrates that kenaf fibres disperse homogeneously within the polymer matrix.

Meanwhile, Figure 3b presents the flexural strength and modulus of kenaf composite–reinforced secondary additive, including CNT and MAPP. The results demonstrate that the addition of a secondary additive improved the mechanical properties of the materials until 46 MPa. It was earlier reported that an additional additive can aid in reducing the particles sizes, which allows for the fillings of voids between the kenaf fibres [36]. These further strengthen the composite materials, as different sizes and shapes of the additive are able to increase the compaction of the materials [6,37]. Figure 4f proved that the produced composite was more homogenous compared to the composite with a single additive (CNT), which supported the flexural strength results obtained. However, as shown in Figure 3b, the flexural strength started to deteriorate as the additive loading went above 3 wt %. The additive tended to agglomerate, while the kenaf fibre tended to disperse ununiformly, leading to poor mechanical properties [6,38]. Also, the weak adhesion of the kenaf composite resulted in low flexural strength due to the hydrophilic surface of the kenaf materials [20]. 

Therefore, studies using the coupling agent to overcome the surface adhesion were performed, and the results are shown in Figure 3c. These results demonstrate that the kenaf/epoxy composite, which is immersed in alkaline solution NaOH, shows lower flexural strength at 100MPa compared to untreated composite at 180 MPa. Theoretically, the treated kenaf composite experiences higher mechanical properties compared to untreated kenaf composite because of the hydrophilicity of the kenaf. It is interesting to note that the contradicting results reported were because the weight loss of NaOH was greater than for the untreated kenaf composite [7,34]. Based on the results obtained, the value of the flexural strength was in opposition to the tensile strength due to the fibre orientation after being treated, as shown in Figure 4d [39,40]. The treated fibre tends to have a smooth fibre surface compared to the untreated fibre, shown in Figure 4c. The better fibre orientation was due to the shear induction, which strengthen the kenaf composite. 

### 3.3. Impact Properties 

**Figure 5:**
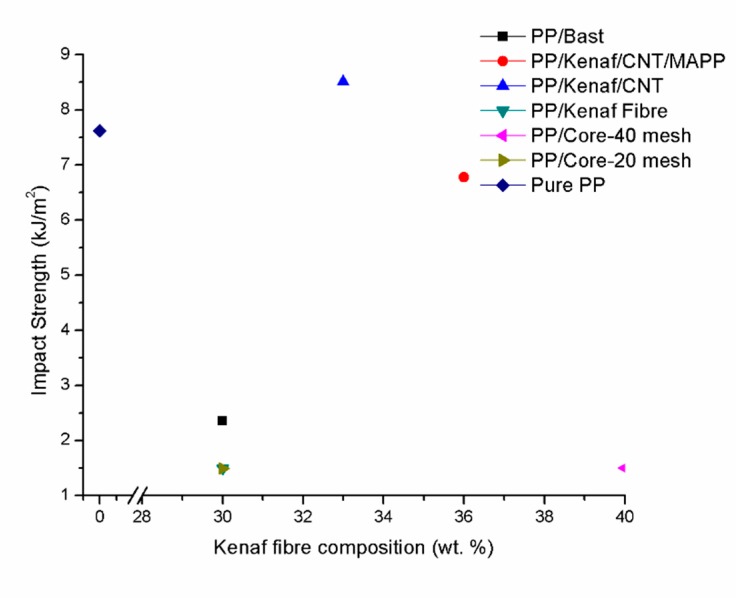
Impact strength analysis.

Impact properties of kenaf composite materials of various type of additive and kenaf fibres are shown in Figure 5. The results indicate that the kenaf composite–reinforced CNT exhibited higher impact strength with 8.5 kJ/m^2^ at 33 wt % of kenaf fibre compared to the addition of CNT and MAPP with 7 kJ/m^2^. According to the results obtained, as the sizes of the additive decreases, the contact resistance between them is thus enhanced, leading to lower mechanical properties [6,10]. 

## 4. Conclusions

In this research, kenaf composites were developed and fabricated at different kenaf fibre loadings, processing methods, and machining. The kenaf composite fabrication involved three types of polymers, which were polypropylene, polylactic acid, and epoxy. Through this process, the kenaf composite made of epoxy resin was the most promising composites materials as it exhibited excellent flexural strength at 180 MPa. The mechanical properties the treated kenaf fibre were better compared to the untreated kenaf fibre. We therefore conclude that alkali treatment can develop a smooth structure and better align the kenaf fibres, which enhances the mechanical properties. These studies indicated that the kenaf composite–reinforced epoxy resin can be considered as an alternative to reinforce materials for the preparation of automotive parts. In our future work, a detail study on flexural strength will be conducted, as kenaf composite is a promising natural fibre used as an alternative in modern technology.
